# Design, Implementation, and Evaluation of a Community-Based Phygital Telemonitoring Program for Older Adults: Multisite Retrospective Pilot Study in Singapore

**DOI:** 10.2196/56905

**Published:** 2025-10-30

**Authors:** Yichi Zhang, Michelle Cheok Yien Law, Soon Keong Wee, Benjamin Sian Teck Lee, Bing Liang Alvin Chew, Wei Peng Teo, Edmund W J Lee

**Affiliations:** 1 Physical Education and Sports Science Department, National Institute of Education, Nanyang Technological University, Singapore, Singapore; 2 Ageing Research Institute for Society and Education, Interdisciplinary Graduate Programme, Nanyang Technological University, Singapore, Singapore; 3 Lee Kong Chian School of Medicine, Nanyang Technological University, Singapore, Singapore; 4 School of Biological Sciences, Nanyang Technological University, Singapore, Singapore; 5 NTU Institute for Health Technologies, Interdisciplinary Graduate Programme, Nanyang Technological University, Singapore, Singapore; 6 Science of Learning in Education Centre, National Institute of Education, Nanyang Technological University, Singapore, Singapore; 7 Department of Media and Communication, City University of Hong Kong, M5010, 5/F Run Run Shaw Creative Media Centre, 18 Tat Hong AvenueHong Kong, China (Hong Kong), +(852) 3442 8868; 8 Institute of Digital Medicine, City University of Hong Kong, Hong Kong, China (Hong Kong)

**Keywords:** telehealth, telemonitoring, aging, health access, community health, technology, health inequalities, digital health, pilot study, implementation

## Abstract

**Background:**

Noncommunicable diseases, particularly hypertension, diabetes, hyperlipidemia, and obesity, are on the rise among older adults in Singapore, emphasizing the need for effective screening, monitoring, and educational interventions. The traditional health care model, relying on in-person visits to review patients, poses risks of underreported cases and missed opportunities for early interventions to manage complications. Community-based telemonitoring programs present promising opportunities to extend telehealth services to underserved populations, thereby mitigating the digital divide and addressing health inequalities.

**Objective:**

This study aimed to retrospectively evaluate the implementation of a community-based telemonitoring program, the Community Telehealth Service (CTS), developed to reduce digital barriers and raise awareness for regular health screening among older adults in the Singapore community. It also aimed to generate insights for scaling up similar telehealth initiatives in the community.

**Methods:**

This retrospective study used the (1) Implementation Research Logic Model, (2) Reach, Effectiveness, Adoption, Implementation, and Maintenance framework, and the Implementation Outcomes Framework to guide the design and evaluation of CTS’ implementation strategies. Outcomes covered implementation outcomes (reach, adoption, feasibility, and cost), service outcomes (safety and preliminary effectiveness), and user outcomes (satisfaction). Data were collected from operational statistics and structured user feedback surveys across 3 phases of implementation at different community sites.

**Results:**

Over the course of the 3 phases, CTS has reached more than 800 older individuals and 147 health ambassadors, with the participation of community organizations, health care institutions, academic collaborators, corporate sponsors, and government agencies. Operational statistics indicated that CTS was delivered consistently across 3 sites, with improving show-up rates and stable service hours. User feedback was generally positive, citing convenience, perceived value, and appreciation for health ambassador support. However, challenges were noted in referral tracking due to differing workflows across partners and in collecting user feedback, particularly in later phases where the survey was perceived as lengthy and complex for older users. Several areas for improvement were identified, such as incorporating more health assessments, providing more health-related information, and improving the referral process.

**Conclusions:**

This early-stage retrospective study suggests that community-based telehealth programs may be a feasible and acceptable approach to delivering preventive services in community settings. While initial findings are promising, further rigorous research is needed to evaluate long-term outcomes, integration with health systems, and potential for scale-up.

## Introduction

The global challenges of aging arise from people living longer and falling birth rates [1], leading to a projected doubling of the population aged 65 years or older from 2021 to 2050 [2]. It is accompanied by a gradual deterioration of physical and mental capacities and increased vulnerability to diseases [3]. Noncommunicable diseases (NCDs), such as cardiovascular diseases, cancers, chronic respiratory diseases, and diabetes, are particularly prevalent among older individuals and account for 74% of all deaths globally [4]. This demographic shift brings about societal challenges from the economy to health care policies [2,3].

Singapore has a rapidly aging population [5] with a surging proportion having multiple NCDs [6-8], and on average, an individual may spend the last 10.6 years of their lives in poor health conditions [3,4,9]. In a survey with 4549 community-dwelling Singapore residents aged 60 years and older, 38% of the respondents perceived their health as fair or poor, and the “3-highs” (high blood pressure or hypertension, high blood cholesterol or hyperlipidemia, and high blood sugar or diabetes) ranked among the top 5 most prevalent self-reported chronic conditions [7]. Obesity has also been observed to have a noticeable increase across all age groups since 2017, particularly among individuals aged 50 to 74 years [8]. This underscores a pressing need for effective screening, monitoring, and educational interventions targeted at this demographic [10-12], so as to pursue diverse interests and make meaningful contributions throughout their lives [2,3].

In the traditional health care model, health professionals could only review their patients during in-person visits, and complications may arise along with missed opportunities for early intervention [13]. This is notwithstanding underreported cases due to low participation in health screening and self-measurement practices, especially the “3-highs” which disproportionately affect the underprivileged [10-12,14]. Furthermore, the COVID-19 pandemic led to the deferment of nonurgent health services, lowered participation in NCD and cancer screenings [15], and the safe management measures might have impacted older individuals’ healthy lifestyle practices [15].

These factors spurred the use of telehealth, which was brought about by the advent of smart health technologies, such as wearable sensors coupled with mobile apps or computer software [16]. It simplified and enhanced the access to taking serial measurements and self-monitoring of physiological parameters outside the traditional health care settings [16]. This has shown potential in providing reliable health data and empowering older individuals, caregivers, and health care professionals alike to create better awareness of one’s vitals, influence health behaviors, and track interventions toward a better overall quality of life [17-19], especially when analyzed in conjunction with individualized reference ranges [20].

Although local health institutes have introduced home-based telemonitoring services to aid patients in monitoring their NCDs [13,21-25], these services may not be readily available to those with low health screening participation or undetected conditions. Furthermore, some barriers associated with this approach may make it less suitable for older adults, such as perceived low usefulness [26,27], concerns over cost [26], challenges in accessing or operating the technologies without sufficient support [27,28], as well as privacy and safety concerns [27,28].

To overcome the limited access to home-based telemonitoring, community-based telemonitoring kiosks have emerged as an alternative. Courtney et al [29] conducted a qualitative study exploring the acceptability and perceived value of such kiosks among older adults and community case workers, and findings highlighted their potential in reducing health disparities and bridging the digital divide. While such kiosks can deliver health services to underserved populations with minimal or no cost [30,31], their success is often hindered by low user engagement [30], stemming from unawareness, uncertainty about their benefits, and lack of motivation [32,33]. This underscores the importance of addressing contextual barriers as well as designing and evaluating tailored implementation strategies early to support successful adoption and scale-up.

This retrospective pilot study aims to evaluate the implementation of a community-based telemonitoring program designed for older adults, Community Telehealth Service (CTS), and to generate insights to inform future scale-up for community-based telehealth programs. In particular, we will answer 2 research questions. First, can such a community-based telehealth program be feasibly implemented to support older adults in real-world settings? Second, what are the key areas of improvement to motivate the uptake of community-based telehealth programs?

## Methods

### Intervention Design

CTS was developed to reduce older adults’ barriers to adopting digital health technologies and to raise their awareness of the importance of regular health monitoring, ultimately increasing the uptake of preventive services. Its design was guided by the Capability, Opportunity, Motivation-Behavior (COM-B) model [34], which emphasizes the interplay of capability, opportunity, and motivation in behavior change. Phygital telehealth kiosks were strategically located in easily accessible community spaces to create physical and social opportunities; trained health ambassadors provided on-site assistance to enhance users’ capability in navigating digital tools; and real-time and personalized health feedback was delivered to strengthen motivation and support long-term engagement with health monitoring behaviors. CTS has been designed not as a replacement but instead as a complement to traditional health care delivery for the older population, by lowering their barriers to accessing personal health monitoring technologies, linking them back to the formal health care system, and extending health care beyond the clinical settings.

### Target Population

While our primary focus was on Singapore residents (including foreigners) aged 50 years and older, we remained open to the younger age groups. Within this broader range, we gave priority to those who were born in or before the 1950s as well as the low-income groups (household monthly income per person lower than S $2000 which is equivalent to US $1473).

#### Health Assessments and Technologies

To tackle the rising prevalence and underreporting of the “3-highs” and obesity rates in Singapore [10-12,14], we integrated a suite of point-of-care assessments to enable early detection and monitoring. These included artificial intelligence–assisted eye screening; cordless upper-arm blood pressure and heart rate monitors; smart scales to measure weight, BMI, and body fat composition; and capillary blood glucose measurement.

#### Phygital Kiosk

To create a safe and private environment for our telemonitoring program, we repurposed the biosafety booths, which were originally designed for mass swabbing exercises [35] (Multimedia Appendix 1). The adaptable modular design of the kiosks allowed us to customize the telemonitoring program according to specific requirements. In addition, the kiosks were equipped with an internet connection that enabled the uploading of health data, printing of health reports, and conducting teleconsultation when necessary.

#### Manpower

At each implementation site, trained health ambassadors were stationed to provide real-time assistance and support to users, particularly those with limited digital or health literacy. These ambassadors helped users navigate the telehealth kiosks, conduct health assessments, and facilitate understanding of their results.

#### Referral Management System

The integration of a referral management system was a pivotal and essential component of our program. Recognizing the importance of seamless coordination and collaboration with health care providers, we established a streamlined procedure to ensure that users in need could be identified and referred to polyclinics and community nurse posts to receive timely, targeted, and subsidized diagnosis and follow-up care.

#### User Journey

**Figure 1.**
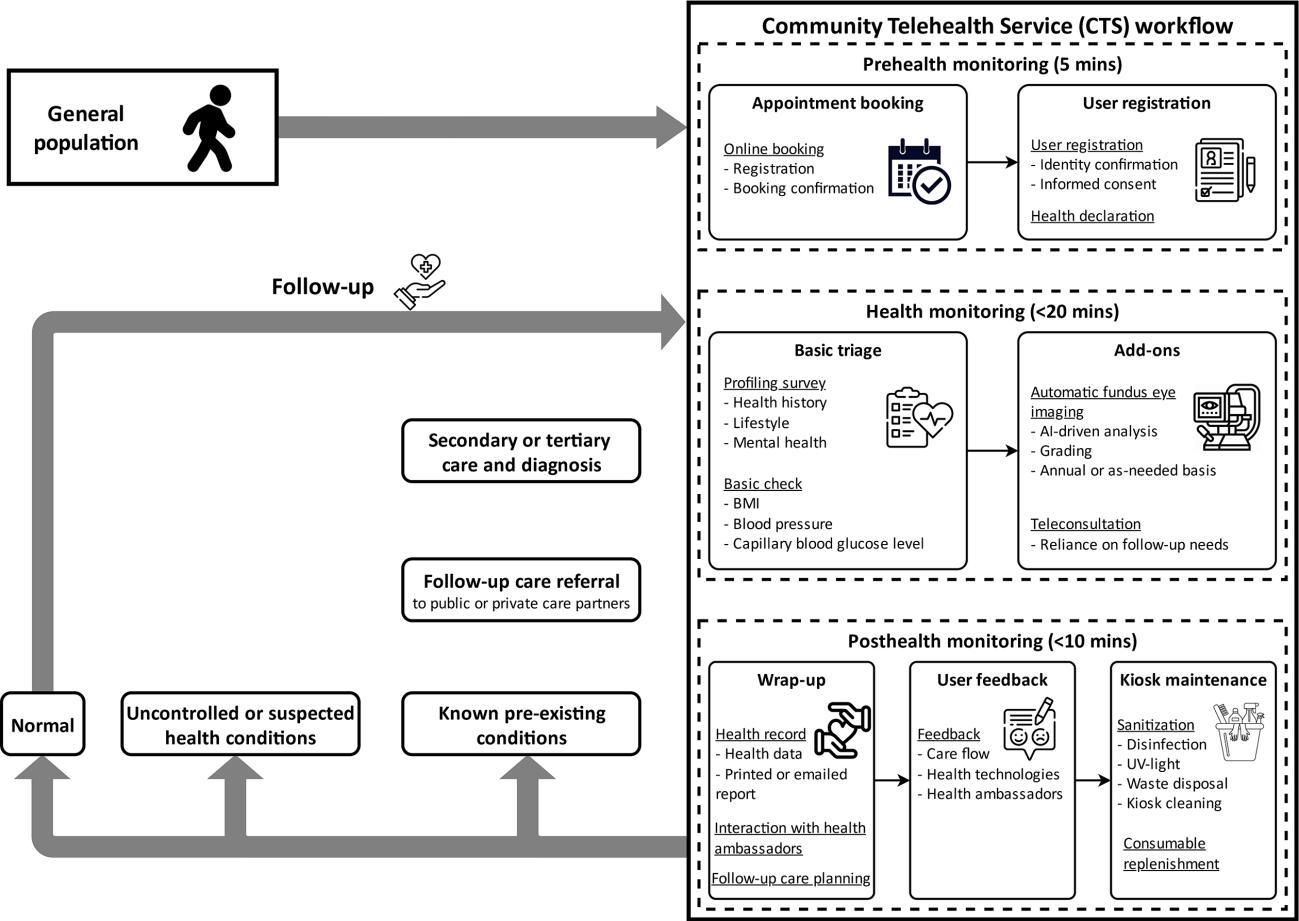
Overview of Community Telehealth Service phygital telehealth user journey.

The user journey in our telemonitoring prototype is illustrated in Figure 1. Health ambassadors would first verify users’ identities using a secure registration system on smart tablets, then guide the users through the profile surveys with health history, various measurements, and communication of the results, as well as any remarks from the users. Users’ eye reports were sent to the Singapore National Eye Center for manual verification, while the other devices had been approved by the Health Sciences Authority and were able to provide immediate results under the operation of trained health ambassadors. In the event of abnormal readings, users would be directed to the referral system for professional diagnosis and follow-up care.

### Determinants and Implementation Strategies

**Figure 2.**
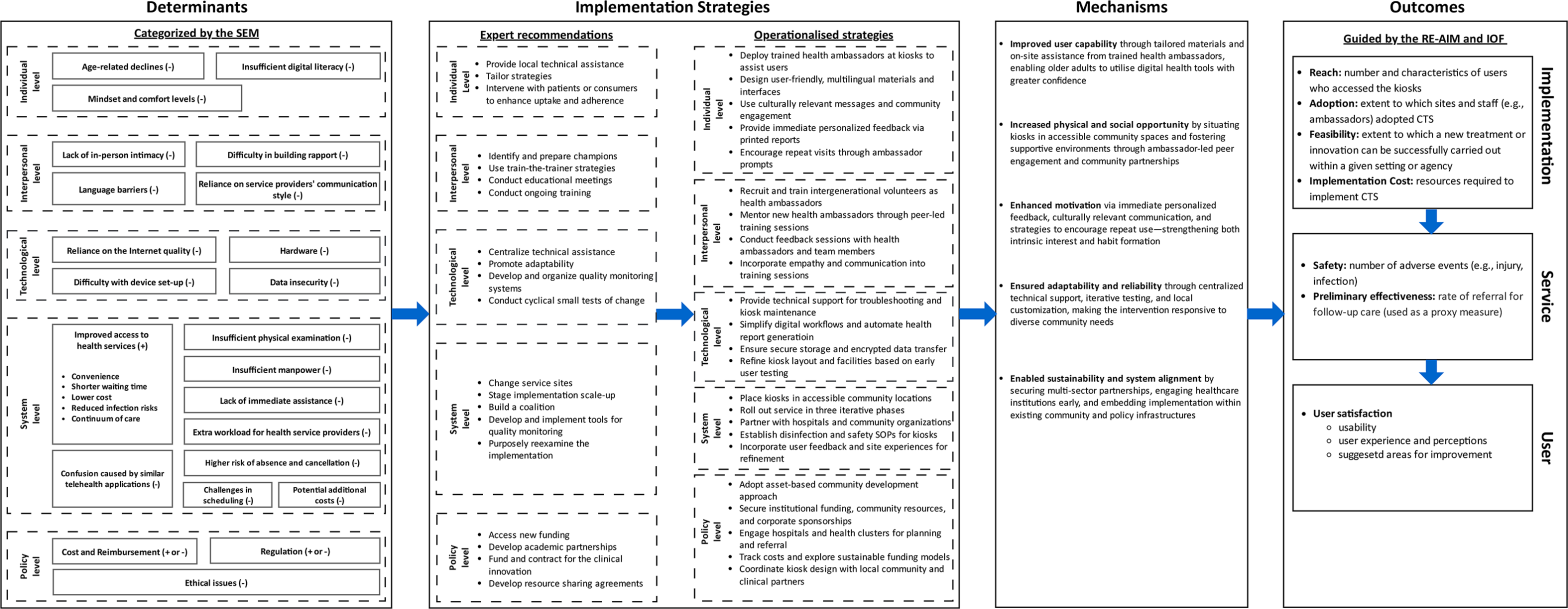
Implementation Research Logic Model to guide the design and evaluation of Community Telehealth Service implementation strategies. IOF: Implementation Outcomes Framework; RE-AIM: Reach, Effectiveness, Adoption, Implementation, and Maintenance Framework; SEM: social ecological model.

The development and evaluation of CTS’ implementation strategies were guided by the Implementation Research Logic Model (IRLM) [36], which provides a structured approach to map the context-specific determinants, Expert Recommendations for Implementing Change compilation (ERIC)−aligned implementation strategies, underlying mechanisms of action, and key outcome measures (Figure 2).

#### Determinants

Facilitators and barriers to telehealth implementation were identified through our scoping review [37] and an earlier local qualitative study involving 15 older adults, 12 health service providers, and 2 telehealth providers. These determinants were organized according to the social ecological model (SEM) [36] across 5 levels, which are individual, interpersonal, technological, system, and policy levels. Key barriers included low digital literacy, difficulty building rapport with telehealth deliverers, reliance on internet connectivity, insufficient physical examination, insufficient manpower to deliver telehealth services, as well as ethical and data security concerns.

#### Implementation Strategies

Based on the determinants identified, we selected a set of implementation strategies from the ERIC taxonomy [38] and operationalized them across the SEM levels to address the multilevel determinants.

At the individual level, the trained health ambassadors provided users with on-site assistance, friendly and multilingual materials, as well as personalized health feedback upon receiving the printed health reports to enhance engagement. At the interpersonal level, ambassadors from different age groups were recruited and trained using a train-the-trainer model that covered not only basic health knowledge and health assessment skills but also empathy and communication techniques. All training materials were reviewed by professional medical advisors to ensure clinical accuracy and alignment with local guidelines. The health ambassadors were paired to comanage each kiosk—typically matching a younger ambassador who was more adept with technical operations with an older ambassador who brought stronger interpersonal communication skills (Multimedia Appendix 2). This intergenerational pairing was intentional to enhance both technical proficiency and user engagement, fostering a warm and age-friendly environment that resonated with older adults. Technological strategies included centralized technical support, simplified digital workflows, and iterative user testing to improve system usability and reliability. At the system level, our telehealth kiosks were deployed in accessible community locations through phased implementation, in collaboration with community, health care, and corporate partners. Safety protocols and feedback loops were established to guide iterative improvements. At the policy level, funding was secured through university, community sources, and corporate sponsorships, while academic partnerships were formed to support planning, training, and sustainability.

### Outcome Evaluation and Data Collection

The evaluation of the CTS implementation strategies was guided by the Reach, Effectiveness, Adoption, Implementation, and Maintenance (RE-AIM) framework [39,40] and complemented by the Implementation Outcomes Framework [41], to assess key domains of implementation performance and user experience in real-world settings. These outcomes were also categorized according to the IRLM into three domains: (1) implementation outcomes, (2) service outcomes, and (3) user outcomes.

Implementation outcomes focused on the extent to which CTS could be successfully executed, including its reach (number of users as well as the number of health screenings and health ambassador training sessions conducted), adoption (number of participating community partners, social service agencies, medical partners, and academic partners), feasibility (number of operating hours, cancellation, and no-show rates), and implementation cost. Service outcomes included safety (the number of adverse events reported) and preliminary effectiveness (the rate of referral for follow-up care as a proxy). Both implementation and service outcomes were collected from the operational statistics.

User outcomes focused on satisfaction, covering CTS usability, user experience with and perceptions of CTS, and suggested areas for improvement collected through structured user feedback surveys. All users were invited to fill out feedback on Google Forms at the conclusion of their screenings, and their participation was encouraged but not mandatory. In phase 1, the feedback survey encompassed 6 short questions to explore users’ experience with appointment booking, rating of the waiting time, satisfaction with health ambassadors’ instructions, overall experience, intention to continue using CTS, and perceptions of the program (Multimedia Appendix 3). In phases 2 and 3, an adapted version of the Telehealth Usability Questionnaire developed by Parmanto et al [42], a robust tool to assess the usability of telehealth implementation and services, was used. The adapted tool included 24 items rated on a 5-point Likert scale, along with an extra open question to cover various usability factors of the enhanced services, such as publicity, registration and waiting time, service reliability, interaction with health ambassadors, perceived usefulness and ease of use, quality of services, satisfaction and future use, and potential areas to improve (Multimedia Appendix 3). In addition to the survey, key areas for improvement were identified through health ambassador feedback and on-site observations during the program implementation and were summarized thematically to inform iterative program refinement.

### Data Analysis

Quantitative data were analyzed using RStudio and presented using descriptive statistics, while qualitative data from open-ended user feedback and on-site observations were analyzed using rapid thematic analysis [43], given that the number of qualitative feedback responses was manageable and could be effectively analyzed manually.

### Ethical Considerations

This project was initially approved and implemented under the Graduate College – Community Engagement Project (CEP), Nanyang Technological University [44], and therefore did not undergo ethics review at the time of user enrollment. At the time of enrollment in CTS, all users provided consent to participate in the program and to allow their anonymous data to be used for analysis. For this retrospective secondary analysis, only fully anonymized data were used, and no additional consent was required. Institutional review board approval was obtained from the University’s Institutional Review Board (IRB-2022-970). The study team had full permission to access and analyze the dataset for research purposes. All procedures adhered to the principles of the Declaration of Helsinki and local regulatory requirements.

## Results

### Phased Implementation

CTS was deployed in 3 phases progressively. In phase 1, the telehealth kiosk was deployed in the Northeast of Singapore daily from mid-January to the end of February 2021, after the circuit breaker (a strict stay-at-home order and cordon sanitaire by the government [45,46]). The aim of this phase was to rapidly collect preliminary user feedback from the users for program enhancement for later phases. Based on user feedback in phase 1 (Table 1), we brought various enhancements to phases 2 and 3. In phase 2, CTS was launched in the West, from end-September 2021 to end-March 2022. Due to the heightened safety measures to slow the spread of COVID-19 [47], the program was temporarily closed for 2 months from October to December 2021. In phase 3, the program was launched in the East in January 2022 and remained operational for a 3-month period until March 2022. User feedback in phases 2 and 3 is summarized in Table 2. Details of the implementation of CTS across 3 phases are summarized in Table 3.

**Table 1.** Summary of user feedback in phase 1.

Item and response	Phase 1 (n=389; response rate=62%), n (%)
Did you register an appointment timeslot by yourself?	
Yes	258 (66.3)
No	131 (33.7)
How do you find the waiting time required from the start of the booth session to receiving your health report?	
Appropriate	324 (83.3)
Too short	42 (10.8)
Too long	23 (5.9)
How do you find the verbal instructions given by the health ambassadors?	
Very clear	311 (79.9)
Clear	55 (14.1)
Acceptable	22 (5.7)
Unclear	1 (0.3)
How do you find the overall CTS^a^ experience?	
Very good	304 (78.1)
Good	70 (18)
Acceptable	14 (3.6)
Not good	1 (0.3)
Overall, do you want to continue seeing and using the CTS booth as a community resource for health monitoring?	
Yes	383 (98.5)
No	6 (1.5)

a CTS: Community Telehealth Service.

**Table 2.** Summary of user feedback in phases 2 and 3.

Item and response	Phase 2 (n=103; response rate=61; 59.2%)		Phase 3 (n=79; response rate=12; 15.2%)	
	Mean (SD)	Mode	Mean (SD)	Mode
Registration and waiting time				
I think the waiting time is appropriate.	4.8 (0.5)	5	4.3 (0.8)	5
Reliability of health monitoring				
I think the visits provided over CTS are the same as in-person visits to the clinics.	4.3 (0.9)	5	3.8 (0.8)	3
When the machine/equipment/monitor reported any error (eg, the glucose meter showed an error message or the eye machine cannot capture eye images), the health ambassador could resolve the issue easily and quickly.	4.4 (0.9)	5	4.1 (0.8)	4
The health ambassadors could clearly tell me the problems with the health indicators shown in the health report.	4.7 (0.7)	5	4.3 (0.7)	5
I have confidence in the health ambassadors’ skills.	4.5 (0.7)	5	4.3 (0.6)	5
I have got sufficient information about my health conditions.	4.6 (0.6)	5	4.0 (0.7)	4
Interaction with health ambassadors				
I feel that the health ambassadors cared about me.	4.8 (0.5)	5	4.3 (0.6)	4
I had enough time to talk and interact with the health ambassadors.	4.7 (0.6)	5	4.3 (0.6)	4
I felt I was able to express myself effectively.	4.6 (0.7)	5	4.3 (0.6)	4
I could hear the volunteers/health ambassadors clearly in CTS.	4.8 (0.5)	5	4.4 (0.6)	5
I feel that the health ambassadors talked to me in a way that was easy to understand.	4.8 (0.5)	5	4.4 (0.6)	5
I felt comfortable communicating with the volunteers/health coaches.	4.8 (0.5)	5	4.4 (0.6)	5
Usefulness and ease of use				
CTS improves my access to healthcare services.	4.6 (0.6)	5	4.3 (0.6)	4
CTS saves me time traveling to a hospital or specialist clinic.	4.6 (0.7)	5	4.4 (0.6)	5
CTS provides for my healthcare needs.	4.4 (0.8)	5	4.1 (0.6)	4
CTS is simple to use.	4.7 (0.7)	5	4.4 (0.6)	5
Quality of service				
My interaction with CTS is pleasant.	4.8 (0.4)	5	4.4 (0.6)	5
I think CTS is well-organized.	4.7 (0.6)	5	4.3 (0.7)	4
I feel that the service that CTS provided is satisfactory.	4.8 (0.5)	5	4.3 (0.7)	5
Satisfaction and future use				
CTS is an acceptable way to receive healthcare services.	4.7 (0.6)	5	4.3 (0.6)	4
I would use CTS again.	4.6 (0.7)	5	4.4 (0.6)	5
Overall, I am satisfied with CTS.	4.7 (0.6)	5	4.3 (0.6)	4

**Table 3.** Details of the implementation of Community Telehealth Service across 3 phases.

Element	Phase 1	Phase 2	Phase 3
Period of deployment	January 9 to February 27, 2021	September 25, 2021, to March 31, 2022 (temporary closure from October to December 2021)	January 3, 2022, to March 31, 2022
Location	Punggol 21 Community Center (Northeast) – located near digital ambassadors who could assist older adults in improving their digital skills	Bukit Batok (West) – located beside the partner senior center	Chai Chee (East) – located near the partner nonprofit charitable health care organization
Operating hours	9 AM to 5 PM daily	Flexible opening hours based on the booking and health ambassadors’ availability with at most 3 days per week	Flexible opening hours based on the booking and health ambassadors’ availability with at most 3 days per week
Health assessments and technologies	Weight BMI Body fat Muscle Visceral fat Blood pressure Heart rate Glucose level Eye health and risk of cardiovascular diseases – sent to the Singapore National Eye Center for manual verification)	Mental health test using WHO-5^a^ Weight BMI Body fat Muscle Visceral fat Blood pressure Heart rate Glucose level Eye health (risks of diabetic retinopathy, glaucoma, and age-related macular degeneration) – automatically generated by the eye machine	Mental health test using WHO-5 Weight BMI Body fat Muscle Visceral fat Blood pressure Heart rate Glucose level Eye health (risks of diabetic retinopathy, glaucoma, and age-related macular degeneration) – automatically generated by the eye machine
Referral management system	Sent those reports with possible abnormalities to Sengkang Hospital Community Nurse Post	Referred users with possible abnormalities to a National University Polyclinic nearest to users’ homes, and included brief notes in the printed health reports	Referred users with possible abnormalities to SATA CommHealth, a non-profit charitable health care organization, and included brief notes in the printed health reports
Publicity	Social media Door-to-door publicity Displaying publicity posters in the lift lobbies Media mentions	Social media Door-to-door publicity Community outreach Roadshow	Social media Door-to-door publicity Community outreach Displaying publicity posters on digital display panels of residential blocks Roadshow

a WHO-5: World Health Organization-Five Well-Being Index.

### Implementation Outcomes

All implementation outcomes are summarized in Table 4.

**Table 4.** Summary of the operational statistics.

Outcome	Phase 1	Phase 2	Phase 3
Total number of operation hours	236 h (health screening and health ambassador training were held concurrently)	243 h – screening 47 h – health ambassadors training	90 h – screening 36 h – health ambassadors training
Total number of screenings conducted	627 screenings: 277 males and 350 females Mean age 57.0 (SD 10.2) years	103 screenings: 30 males and 68 females (5 returning users) Mean age 65.0 (SD 9.5) years	79 screenings: 38 males (3 returning users) and 37 females (1 returning user) Mean age 68.0 (SD 9.2) years
Cancellation and no-show rates	About 26.8%^a^	About 15.6%^a^	About 11.2%^a^
Referral rates	26%	Untracked	Untracked
Total number of volunteers trained	55 student health ambassadors 11 community health ambassadors 45 more volunteers on the waitlist	30 student health ambassadors^b^ 51 community health ambassadors (excluding health ambassadors trained at phase 1)^b^	30 student health ambassadors^b^ 51 community health ambassadors (excluding health ambassadors trained at phase 1)^b^
Implementation cost	S $19,884.73 (US $15,423.31)	S $3806.24 (US $2952.26)^c^	S $3806.24 (US $2952.26)^c^

a Some walk-in visitors may have requested partial screenings that were not accounted for in the registration system.

b The numbers of student and community health ambassadors (n=30 and n=51, respectively) trained is the total number in both phases 2 and 3 instead of each phase.

c The total cost for phases 2 and 3 is S $3806.24 (US $2952.26).

#### Reach

Over the 8 weeks in phase 1, a total of 627 screenings for 277 males and 350 females (mean age 57.0, SD 10.2 y) were conducted and 66 health ambassadors were trained, while another 45 registered volunteers were waiting for training. Phase 2 completed 103 screenings (30 males, 68 females, mean age 65.0, SD 9.5 y, of whom 5 were returning users. Phase 3 reached 79 users (38 males, 37 females, mean age 68.0, SD 9.2 y), including a total of 4 returning users.

Across all 3 phases, similar recruitment strategies were used, although media release proved most effective in phase 1. In phase 2 (n=103, 61 responses were received with a response rate of 59.2%), most users learned about CTS through publicity materials (n=22, 36.1%) and door-to-door publicity (n=15, 24.6%), while others cited passing-by (n=10, 16.4%), social media (n=8, 13.1%), and referrals from their social network (n=6, 9.8%). Over half (n=34, 55.7%) registered independently, and those who received assistance were mainly supported by health ambassadors (n=15, 55.6%), followed by family members or helpers (n=9, 33.3%) and community center staff (n=3, 11.1%). In phase 3 (n=79, 12 responses were received with a response rate of 15.2%), awareness was primarily through passing-by (n=5, 41.7%) and door-to-door publicity (n=4, 33.3%), with fewer citing publicity materials (n=3, 25%). Fewer registered independently (n=5, 41.7%), and all assisted registrations were supported by health ambassadors.

#### Adoption

During phase 1, partnerships were established with 4 community partners, 2 medical partners, and 6 corporate partners. Funding was received from 5 sources, including research grants, university CEP funding, as well as corporate sponsorships. As the service progressed into phases 2 and 3, partnerships expanded to 6 grantors, 10 government and community partners, 2 medical partners, and 8 corporate sponsors. In addition, an additional academic partnership was established.

#### Feasibility

CTS operations in phase 1 spanned 236 hours, during which health screenings and ambassador training were conducted concurrently. The cancellation or no-show rate was approximately 26.8%. In phase 2, the program operated for a total of 290 hours (243 h for screening and 47 h for training), with a no-show rate of 15.6%. Phase 3 recorded 126 total operating hours (90 h for screening and 36 h for training), with a cancellation or no-show rate of 11.2%.

#### Implementation Cost

The implementation costs for all 3 phases were estimated based on the sponsorship with actual claims made to research funding and university CEP funding. The implementation costs for phase 1 and phases 2 and 3 were S $19,884.73 (US $14,913.55) and S $3806.24 (US $2809.01), respectively.

### Service Outcome

#### Safety

The project remained free of adverse events throughout all 3 phases, with no occurrences of COVID-19 infections, injuries, or data breaches reported. This absence of incidents underscores the effectiveness of the safety protocols and the efforts of all stakeholders in maintaining a secure and safe environment.

#### Preliminary Effectiveness – Referral Rate

We referred 163 (26%) users to the community nurse post and Singapore National Eye Center for follow-up care in phase I. We failed to consistently track referral rates in phases 2 and 3, as the medical partners involved operated under distinct referral workflows and documentation systems. In addition, a resurgence of COVID-19 cases during these 2 phases caused interruptions in referral processes, particularly affecting coordination between community sites and health care providers. These variations made it difficult to obtain a standardized and complete record of referral outcomes across both locations.

### User Outcome – Satisfaction

#### Usability

In phase 1, we received a total of 389 user feedback responses (response rate=62%), and the responses were predominantly positive (Table 1). In addition, 66.3% (258/389) of users were able to book an appointment independently and the rest required some assistance from either their social networks or our health ambassadors. Furthermore, 83.3% (324/389) of the users were satisfied with the waiting time. Moreover, 94.1% (366/389) of the users were happy with the waiting time, 99.7% (388/389) of the users found the verbal instructions given by the health ambassadors satisfactory, and 99.7% (388/389) expressed overall satisfaction with the experience. In general, 98.5% (383/389) of the users expressed interest in continuing to use the CTS as a community resource for health monitoring.

In phases 2 and 3, a total of 73 user feedback responses were collected (61 from phase 2 and 12 from phase 3). Overall, users expressed high satisfaction and perceived CTS as a usable service. However, response rates were lower compared with phase 1, which several users attributed to the length and complexity of the questionnaire, particularly challenging for older users. Table 2 presents the survey items along with a summary of the subscale scores.

#### User Experience With and Perceptions of CTS

User feedback from phase 1 reflected strongly positive perceptions of CTS as a health initiative tailored for older adults. Many users appreciated the convenience of receiving multiple health screenings in one location:

*Very convenient. All tests are done at the same spot without the hassle of moving from room to room. [It is] good especially during this COVID-19 times.*

Users also described the service as smooth and efficient and praised the professionalism and warmth of the health ambassadors, for example:

*I am happy [that] we have fellow Singaporeans with the heart to serve the nation. They are doing a good job.*

Several users appreciated the benefits of such community-based telehealth kiosks for the older population and expressed hope that CTS would be expanded to other regions, calling it:

*I am very glad this event was organized for the benefit of the Singapore seniors. I hope this will be a regular event for other parts of Singapore as well…*

#### Suggested Areas for Improvement

While CTS received a high level of acceptability across all 3 phases, user feedback also highlighted potential areas for improvement, for instance, simplifying the registration process, enhancing the environment inside the kiosk and at the waiting areas, and improving the clarity of health reports and publicity materials. A summary of these suggestions and representative quotes is provided in Table 5. Building upon the insights collected from phase 1, enhancements were implemented in phases 2 and 3, such as (1) assigning health ambassadors to timely answer users’ inquiries via WhatsApp (Meta) messages; (2) streamlining the registration process, enhancing the user-friendliness of the registration platform, and sending booking confirmations and reminders through SMS text messages; (3) translating the publicity materials into not only Chinese but also Malay and Tamil; (4) involving medical students and health professionals to enhance the abnormality detection algorithms by taking possible age-specific reference ranges into consideration; and (5) improving the physical environment by providing fans both inside and outside the telehealth kiosks, as well as increasing the seating capacity in the waiting area.

**Table 5.** Suggested areas for improvement.

Areas for improvement	Quotes
Simpler registration and booking confirmation, especially for the older adults	“Give an immediate confirmation of registration for an easy note of the appointment date.” “The date of birth cannot be selected effectively.” “Registration may be difficult for some elderly.”
Enhancing the environment inside the kiosk and in the waiting area	“Prepare some signs to take a seat…” “Cooler aircon preferred…” “One thing that could be improved is the space. Currently is a bit too tight to conduct the check-up.”
Service scale-up (eg, offering additional health assessments, extending operations to more locations, conducting screenings on a regular basis, lengthening service operating hours, and providing support in multiple languages to better serve diverse communities)	“…Wish that there could be more other tests incorporated with this…” “Should extend to more regions as this is very convenient to the seniors.” “If [you could] have one year check-up [,then it] will be better.” “…I hope this will be a regular event for other parts of Singapore as well…” “Appreciate availability of dialect-speaking volunteers”
Improving health reports (eg, including reference ranges in the printed reports and having results reviewed or explained by qualified health care professionals to enhance clarity and credibility)	“Include normal range in the report for comparison” “…and have certified medical personnel/doctors to go through the result of the test…”

In addition to these readily achievable improvements, three more popular suggestions were shared via the health ambassadors: (1) extending the current spectrum of health assessments, (2) providing additional educational health information, and (3) upgrading the current referral management system.

## Discussion

### Principal Findings

The rapidly growing aging population, coupled with the rising prevalence of NCDs and mental health conditions, places escalating pressures on the traditional health care delivery model, rendering disease-centric approaches insufficient and unsustainable in the long run [48,49]. This calls for a paradigm shift toward preventive care [50-52], the creation of new health care infrastructure [50,53-55], the expansion of social and long-term care services within the community [50,56-59], as well as the adoption of technologies to support aging in place [60-62].

To our best knowledge, CTS was the first community-based telehealth program implemented in Singapore. Our CTS is designed to complement the traditional health care model by lowering barriers to accessing personal health monitoring technologies, extending the reach of health care beyond the clinical settings, and establishing a connection between the older population and the formal health care system. Building upon community-based telehealth programs in existing literature, the design, implementation, and evaluation of CTS were guided by IRLM, incorporating different contextual determinants and corresponding implementation strategies identified by our earlier review, qualitative studies, as well as expert recommendations (ERIC). This comprehensive approach aims to improve the rigor and reproducibility of such community-based telehealth services and address the gaps in older individuals’ health-seeking behavior while mitigating health inequity. Findings from this pilot study demonstrate the potential of CTS within the Singapore community and provide insights to pave the way for more comprehensive community-based telehealth programs.

### Potential Areas for Improvement

Based on the user feedback collected, one of the most anticipated improvements is the inclusion of additional health assessments. Advancements in technology have made it possible to incorporate point-of-care tests, such as urine dipsticks, for remote patient monitoring, even by nonmedical individuals [63]. Furthermore, future enhancements may involve integrating physical performance and muscle strength tests (eg, handgrip strength) and cognitive assessments. Nonetheless, additional research efforts are needed to validate these tests when administered by trained nonmedical personnel to a specific subgroup of the population in nonclinical settings.

The second important area for improvement is the provision of more health-related information, which also aligns with users’ interest in a study by Zhang et al [30]. Besides, we also observed a considerable proportion of users who, despite having borderline health conditions or being diagnosed with NCDs, opted out of health screenings, refused medication, or discontinued follow-up care. Guided by these insights, we will work toward implementing different health promotion interventions targeted at empowering older adults to take control of their health journeys and encourage proactive engagement in decisions that impact their overall well-being [64].

The referral management system was observed as another opportunity for improvement. During phase I, we encountered some delays, primarily attributed to our reliance on medical partners to initiate contact with users as well as their increased workload amid the pandemic. As we transitioned into phases 2 and 3 where users were given full autonomy to make decisions, new challenges surfaced: some users refused the referrals, and it was difficult to track their progress. These experiences underscore the importance of establishing efficient and timely communication channels with medical partners to ensure the seamless provision of quality care across the care continuum. Furthermore, robust strategies should be also devised to effectively engage older people and maintain their engagement within the care continuum, contributing to better overall well-being within the community.

### Lessons Learned

#### Human Element in Community-Based Telehealth Programs

Technology advancements have enabled individuals to remotely monitor their physiological parameters outside the clinical settings and seek actionable insights for improving health outcomes [65,66]. Besides income, education, employment, and environment, digital technology has emerged as a new key determinant of health [67,68]. It is noteworthy that while digital health interventions are intended to reduce health inequalities by extending access to health services and addressing unmet health needs through personalized care, there is a concern that they might unintentionally exacerbate health inequities to some extent [69-71]. Therefore, digital inclusion–informed strategies are needed in designing community-based telehealth programs.

In response, we adopted a “phygital” approach, combining both digital and physical elements. While we acknowledge that the implementation of the health ambassador scheme in CTS may limit the generalizability of our findings to other community-based telehealth programs that lack or have minimal human support, as discussed in a study by Zhang et al [30], this approach has the potential to bring significant value to telehealth program delivery. For example, a study by Courtney et al [29] raised several concerns, such as older adults overreacting to minor abnormalities in the health data, the potential risk of using the kiosk as a substitute for regular or urgent health care services, and the false reassurance of blood pressure alone being a comprehensive indicator of overall health status.

In CTS, our well-trained health ambassadors could play a crucial role in mitigating these concerns. Besides conducting health measurements and generating health reports, they assisted users in explaining their measurements based on local clinical guidelines and taking notes of some remarks (eg, their perceived health and self-observed individualized reference ranges) in the reports. This combination of subjective perceptions and objective measurements enhances the delivery of more person-centered care [72]. In addition, health ambassadors could offer actionable advice for lifestyle modifications to positively impact health outcomes; they can also actively encourage all users to undergo regular health screenings at medical institutions and refer them to appropriate medical partners for follow-up care when necessary. Such a comprehensive approach serves to not only minimize the potential misinterpretation of health data but also foster a proactive mindset toward maintaining overall health and well-being.

#### Data Integration Remains a Challenge in Community-Based Telehealth Programs

Community-based telehealth programs allow users to regularly monitor health at community-based facilities, and thus generate a comprehensive record of their health data. The integration of these diverse objective health data points, subjective self-perceived health status, potential individualized reference range, and lifestyles, together with users’ participation in community events and health promotion activities, could support primary care providers in forming a more complete and personalized understanding of an older individual’s health. This, in turn, enables the delivery of more effective, customized, and equitable health care services.

However, challenges arise as these data often remain disconnected from primary care records, possibly due to data inconsistency, lack of standardized data handling and ownership protocols, concerns of breaching data privacy and confidentiality, as well as the risk of generating inaccurate or incomplete data [73,74]. Addressing these gaps and establishing mechanisms to bridge them could be a dedicated effort moving forward.

### Limitations and Future Research

This study has several limitations. First, as a retrospective implementation study, CTS was not originally designed or executed as a research project, which limited the consistency and completeness of some data sources, as well as rigor in research design. For example, although qualitative feedback from users and health ambassadors provided valuable insights, not all feedback was formally documented through audio recordings or transcripts. Some themes were based on on-site observations and informal input, limiting the ability to include original quotes. Building on the promising findings, future research will adopt prospective and rigorous study designs, well-defined eligibility and screening processes, well-designed data collection procedures, and clearly defined implementation protocols to ensure standardized and systematic evaluations.

Second, the response rate for user satisfaction surveys in phases 2 and 3 was low. Several older users reported that the length and complexity of the questionnaire made it challenging to complete, reflecting a common challenge in real-world implementation settings where data collection must balance evaluation needs with user burden. Unlike controlled research environments, community-based programs have to accommodate diverse literacy and cognitive needs, especially among older populations. To improve data quality and inclusivity in future implementations, feedback tools could be simplified so that they are more user-friendly and context-appropriate.

Third, while CTS received multilevel approvals before its implementation, formal inputs from key stakeholders were not systematically captured during the evaluation. Stakeholder perspectives—including those of community partners, social service providers, health care professionals, and policymakers—are critical for understanding contextual enablers, aligning goals, and identifying potential barriers to scale-up. Future studies should incorporate structured stakeholder engagement as part of the scale-up and evaluation process to inform implementation refinement, ensure policy relevance, and strengthen the long-term sustainability and integration of community-based telehealth models.

Finally, although this study was guided by the RE-AIM framework, we did not assess CTS’ clinical effectiveness and maintenance, as it was implemented as a time-limited, early-stage pilot program. Future research will explore the longer-term impact of such community-based telehealth programs on users’ health outcomes, behavioral outcomes, as well as users' maintained engagement over time. To advance this work, a hybrid type 2 effectiveness-implementation study could be used to simultaneously evaluate the clinical effectiveness of CTS while further refining and testing its implementation strategies in real-world settings. In addition, exploring how these programs can be integrated with existing health care systems and national preventive health strategies will be essential to understanding their scalability, sustainability, and broader public health value.

### Conclusions

This retrospective study demonstrated the potential of implementing a community-based telemonitoring program for older adults in Singapore, which combines accessible physical infrastructure with digital health tools and on-site human support. While the early results are promising, further rigorous research is needed to evaluate long-term outcomes, system integration, and scalability. These findings offer valuable insights for designing equitable, user-centered telehealth interventions in community settings.

## Supplementary material

10.2196/56905 Multimedia Appendix 1 Original biosafe booth designed for mass swabbing exercises (left) and the repurposed booth (right).

10.2196/56905 Multimedia Appendix 2 Photos of a health ambassador pair (photos were taken by Caroline Chia / WhatAreYouDoing.sg).

10.2196/56905 Multimedia Appendix 3 User feedback survey questions in phases 1-3.

## Abbreviations

CEP: Community Engagement Project
COM-B: Capability, Opportunity, Motivation-Behavior
CTS: Community Telehealth Service
ERIC: Expert Recommendations for Implementing Change compilation
IRLM: Implementation Research Logic Model
NCD: noncommunicable disease
RE-AIM: Reach, Effectiveness, Adoption, Implementation, and Maintenance
SEM: social ecological model

## References

[1] (2010). Ageing: global population. World Health Organization.

[2] United Nations Department of Economic and Social Affairs (2023). UNDESA world social report 2023: leaving no one behind in an ageing world. https://desapublications.un.org/file/1087/download.

[3] (2021). Ageing and health. World Health Organization.

[4] (2022). Noncommunicable diseases. World Health Organization.

[5] (2022). Population in brief 2022. https://www.strategygroup.gov.sg/files/media-centre/publications/population-in-brief-2022.pdf.

[6] Choo F (2019). Proportion of older adults with multiple chronic diseases surges. SingHealth.

[7] (2019). THE SIGNS study by Duke-NUS researchers identify factors affecting active and productive ageing among older singaporeans. Duke-NUS Medical School.

[8] Ministry of Health Singapore (2020). National population health survey.

[9] (2019). The burden of disease in singapore, 1990 – 2017: an overview of the global burden of disease study 2017 results. https://www.healthdata.org/sites/default/files/files/policy_report/2019/GBD_2017_Singapore_Report.pdf.

[10] Seow LSE, Subramaniam M, Abdin E, Vaingankar JA, Chong SA (2015). Hypertension and its associated risks among Singapore elderly residential population. Journal of Clinical Gerontology and Geriatrics.

[11] Man REK, Gan AHW, Fenwick EK (2019). Prevalence, determinants and association of unawareness of diabetes, hypertension and hypercholesterolemia with poor disease control in a multi-ethnic Asian population without cardiovascular disease. Popul Health Metr.

[12] (2011). Singapore diabetes report 2000 — 2050. International Diabetes Federation.

[13] Sin DYE, Guo X, Yong DWW (2020). Assessment of willingness to tele-monitoring interventions in patients with type 2 diabetes and/or hypertension in the public primary healthcare setting. BMC Med Inform Decis Mak.

[14] Ministry of Health Singapore (2004). National health survey.

[15] (2021). National population health survey 2021 (household interview). Ministry of Health Singapore.

[16] Scott J (2022). How medical devices work with mhealth apps. HealthTech.

[17] McBain H, Shipley M, Newman S (2015). The impact of self-monitoring in chronic illness on healthcare utilisation: a systematic review of reviews. BMC Health Serv Res.

[18] Huygens MWJ, Swinkels ICS, de Jong JD (2017). Self-monitoring of health data by patients with a chronic disease: does disease controllability matter?. BMC Fam Pract.

[19] Paré G, Jaana M, Sicotte C (2007). Systematic review of home telemonitoring for chronic diseases: the evidence base. J Am Med Inform Assoc.

[20] Chester JG, Rudolph JL (2011). Vital signs in older patients: age-related changes. J Am Med Dir Assoc.

[21] Teo J (2020). Polyclinics roll out telehealth service to track blood pressure. SingHealth.

[22] (2021). Ambulatory blood pressure watch monitoring - conditions & treatments. SingHealth.

[23] Primary Tech-Enhanced Care (PTEC). National University Polyclinics.

[24] (2023). Continuous glucose monitoring: a useful tool for diabetes management in primary care. SingHealth.

[25] Flash glucose monitoring. National University Hospital.

[26] Korkmaz Yaylagul N, Kirisik H, Bernardo J (2022). Trends in telecare use among community-dwelling older adults: a scoping review. Int J Environ Res Public Health.

[27] Zhang Y, Lee EWJ, Teo WP (2023). Health-seeking behavior and its associated technology use: interview study among community-dwelling older adults. JMIR Aging.

[28] Goldberg EM, Lin MP, Burke LG, Jiménez FN, Davoodi NM, Merchant RC (2022). Perspectives on telehealth for older adults during the COVID-19 pandemic using the quadruple aim: interviews with 48 physicians. BMC Geriatr.

[29] Courtney KL, Lingler JH, Mecca LP (2010). Older adults’ and case managers’ initial impressions of community-based telehealth kiosks. Res Gerontol Nurs.

[30] Zhang Z, Henley T, Schiaffino M (2022). Older adults’ perceptions of community-based telehealth wellness programs: a qualitative study. Inform Health Soc Care.

[31] Schiaffino MK, Zhang Z, Sachs D, Migliaccio J, Huh-Yoo J (2021). Predictors of retention for community-based telehealth programs: a study of the Telehealth Intervention Program for Seniors (TIPS). AMIA Annu Symp Proc.

[32] Hsieh HL, Tsai CH, Chih WH, Lin HH (2015). Factors affecting success of an integrated community-based telehealth system. Technol Health Care.

[33] Hovey L, Kaylor MB, Alwan M, Resnick HE (2011). Community-based telemonitoring for hypertension management: practical challenges and potential solutions. Telemed J E Health.

[34] West R, Michie S (2020). A brief introduction to the COM-b model of behaviour and the PRIME theory of motivation. Qeios.

[35] Mass screening swab booth. Esco Aster.

[36] Smith JD, Li DH, Rafferty MR (2020). The implementation research logic model: a method for planning, executing, reporting, and synthesizing implementation projects. Implement Sci.

[37] Zhang Y, Leuk JSP, Teo WP (2023). Domains, feasibility, effectiveness, cost, and acceptability of telehealth in aging care: scoping review of systematic reviews. JMIR Aging.

[38] Powell BJ, Waltz TJ, Chinman MJ (2015). A refined compilation of implementation strategies: results from the Expert Recommendations for Implementing Change (ERIC) project. Implement Sci.

[39] Glasgow RE, Vogt TM, Boles SM (1999). Evaluating the public health impact of health promotion interventions: the RE-AIM framework. Am J Public Health.

[40] Glasgow RE, Harden SM, Gaglio B (2019). RE-AIM planning and evaluation framework: adapting to new science and practice with a 20-year review. Front Public Health.

[41] Proctor E, Silmere H, Raghavan R (2011). Outcomes for implementation research: conceptual distinctions, measurement challenges, and research agenda. Adm Policy Ment Health.

[42] Parmanto B, Lewis AN, Graham KM, Bertolet MH (2016). Development of the Telehealth Usability Questionnaire (TUQ). Int J Telerehabil.

[43] Gale RC, Wu J, Erhardt T (2019). Comparison of rapid vs in-depth qualitative analytic methods from a process evaluation of academic detailing in the Veterans Health Administration. Implement Sci.

[44] Graduate college - community engagement project. Nanyang Technological University, Singapore.

[45] COVID-19 pandemic in Singapore - wikipedia. Wikipedia.

[46] (2024). 2020–21 Singapore circuit breaker measures. Wikipedia.

[47] (2021). Resuming our transition towards covid resilience. Ministry of Health Singapore.

[48] Nurjono M, Shrestha P, Ang IYH (2020). Shifting care from hospital to community, a strategy to integrate care in Singapore: process evaluation of implementation fidelity. BMC Health Serv Res.

[49] He AJ, Tang VFY (2021). Integration of health services for the elderly in Asia: a scoping review of Hong Kong, Singapore, Malaysia, Indonesia. Health Policy.

[50] Loong PLH (2023). PM Lee Hsien Loong at the ‘Singapore Ageing: Issues and Challenges Ahead’ book launch. Prime Minister’s Office Singapore.

[51] Eliasen B, Teare H, Vrihenhoek T (2021). Shifting to preventive care in a new health system. HIMSS.

[52] (2022). Three ways digital transformation is accelerating the shift to preventive care. Philips.

[53] High KP (2014). Infrastructure and resources for an aging population: embracing complexity in translational research. Transl Res.

[54] Farrugia G (2023). Healthcare infrastructure needs updating for the digital age. World Economic Forum.

[55] Zalizan T (2022). Next healthcare reform involves helping elderly grow old in community, not assuming all will get frail: ong ye kung - TODAY. TodayOnline.

[56] Ministry of Health Singapore, Ministerial Committee on Ageing (2023). Living life to the fullest: 2023 action plan for successful ageing.

[57] Matsuda S, Yamamoto M (2001). Long-term care insurance and integrated care for the aged in Japan. Int J Integr Care.

[58] He AJ, Chou KL (2019). Long-term care service needs and planning for the future: a study of middle-aged and older adults in Hong Kong. Ageing Soc.

[59] Jing T, Zhao X, Xing H, Bao C, Zhan L (2023). The influence of strong social ties on the choice of long-term care model for middle-aged and older adults in China. Front Public Health.

[60] Wang S, Bolling K, Mao W (2019). Technology to support aging in place: older adults’ perspectives. Healthcare (Basel).

[61] Ollevier A, Aguiar G, Palomino M, Simpelaere IS (2020). How can technology support ageing in place in healthy older adults? A systematic review. Public Health Rev.

[62] Sumner J, Chong LS, Bundele A, Wei Lim Y (2021). Co-designing technology for aging in place: a systematic review. Gerontologist.

[63] (2021). Point-of-care testing. Testing.

[64] World Health Organization (1998). Health promotion glossary.

[65] Majumder S, Mondal T, Deen MJ (2017). Wearable sensors for remote health monitoring. Sensors (Basel).

[66] Gandy K, Schmaderer M, Szema A (2021). Remote patient monitoring: a promising digital health frontier.

[67] (2021). Digital technologies: a new determinant of health. Lancet Digit Health.

[68] Governing Health Futures 2030 Commission (2021). Policy brief: the digital determinants of health.

[69] Yao R, Zhang W, Evans R, Cao G, Rui T, Shen L (2022). Inequities in health care services caused by the adoption of digital health technologies: scoping review. J Med Internet Res.

[70] Richardson S, Lawrence K, Schoenthaler AM, Mann D (2022). A framework for digital health equity. NPJ Digit Med.

[71] Ladin K, Porteny T, Perugini JM (2021). Perceptions of telehealth vs in-person visits among older adults with advanced kidney disease, care partners, and clinicians. JAMA Netw Open.

[72] Shiraz F, Hildon ZLJ, Vrijhoef HJM (2020). Exploring the perceptions of the ageing experience in Singaporean older adults: a qualitative study. J Cross Cult Gerontol.

[73] Guide to healthcare data integration (best practices). True North.

[74] Dankar FK (2023). Practices and challenges in clinical data sharing. arXiv.

