# Supplementary material for: Design, Implementation, and Evaluation of a Community-Based Phygital Telemonitoring Program for Older Adults: Multisite Retrospective Pilot Study in Singapore
Source: JMIR Form Res. 2025 Oct 30;9:e56905. doi: 10.2196/56905 (PMC12574743; doi:10.2196/56905)
Supplement: Multimedia Appendix 1 [file formative-v9-e56905-s001.docx]

# **Multimedia Appendix 1**

| 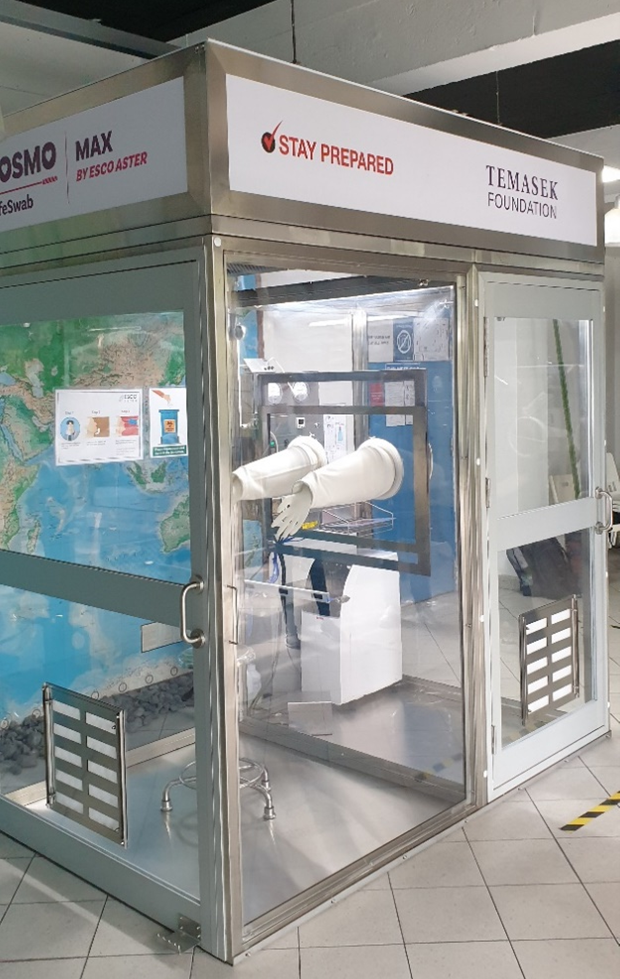 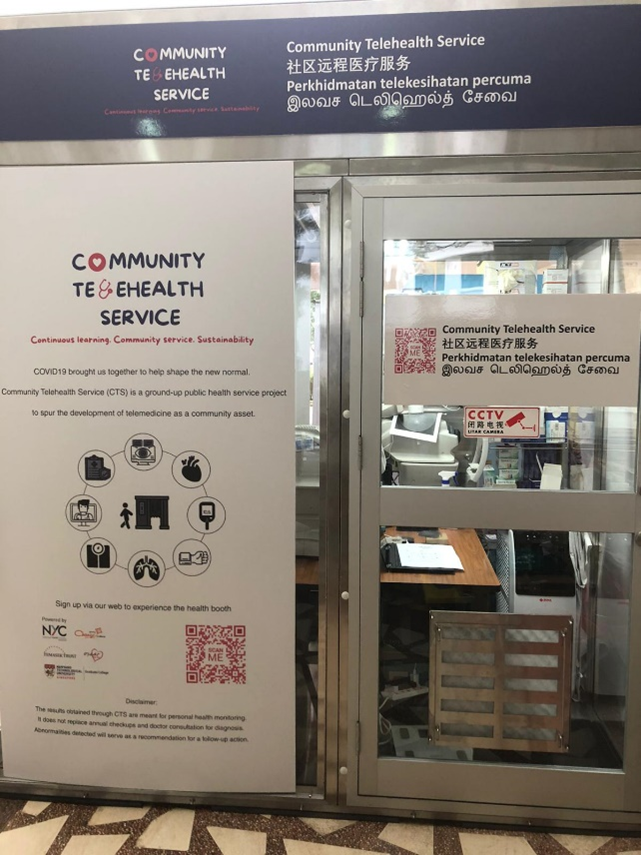 |
| --- |
| Multimedia Appendix 1. Original biosafe booth designed for mass swabbing exercises (left)^35^ and the repurposed booth (right) |
