# Supplementary material for: Design, Implementation, and Evaluation of a Community-Based Phygital Telemonitoring Program for Older Adults: Multisite Retrospective Pilot Study in Singapore
Source: JMIR Form Res. 2025 Oct 30;9:e56905. doi: 10.2196/56905 (PMC12574743; doi:10.2196/56905)
Supplement: Multimedia Appendix 3 [file formative-v9-e56905-s003.docx]

Multimedia Appendix 3

User feedback survey questions in phases 1-3.

| **Phase 1**   - Did you register an appointment timeslot by yourself?   - Yes   - No - How do you find the waiting time required from the start of the booth session to receiving your health report?   - Appropriate   - Too short   - Too long - How do you find the verbal instructions given by the health ambassadors?   - Very clear   - Clear   - Acceptable   - Unclear - How do you find the overall CTS (Community Telehealth Service) experience?   - Very good   - Good   - Acceptable   - Not good - Overall, do you want to continue seeing and using the CTS booth as a community resource for health monitoring?   - Yes   - No   **Phases 2 and 3**   - Registration and waiting time   - I think the waiting time is appropriate. - Reliability of health monitoring   - I think the visits provided over CTS are the same as in-person visits to the clinics.   - When the machine/equipment/monitor reported any error (eg, the glucose meter showed an error message or the eye machine cannot capture eye images), the health ambassador could resolve the issue easily and quickly.   - The health ambassadors could clearly tell me the problems with the health indicators shown in the health report.   - I have confidence in the health ambassadors’ skills.   - I have got sufficient information about my health conditions. - Interaction with health ambassadors   - I feel that the health ambassadors cared about me.   - I had enough time to talk and interact with the health ambassadors.   - I felt I was able to express myself effectively.   - I could hear the volunteers/health ambassadors clearly in CTS.   - I feel that the health ambassadors talked to me in a way that was easy to understand.   - I felt comfortable communicating with the volunteers/health coaches. - Usefulness and ease of use   - CTS improves my access to healthcare services.   - CTS saves me time traveling to a hospital or specialist clinic.   - CTS provides for my healthcare needs.   - CTS is simple to use. - Quality of service   - My interaction with CTS is pleasant.   - I think CTS is well-organized.   - I feel that the service that CTS provided is satisfactory. - Satisfaction and future use   - CTS is an acceptable way to receive healthcare services.   - I would use CTS again.   - Overall, I am satisfied with CTS. |
| --- |
